# Genome-Wide Association Study Unravels *LRK1* as a Dark Respiration Regulator in Rice (*Oryza sativa* L.)

**DOI:** 10.3390/ijms21144930

**Published:** 2020-07-13

**Authors:** Mingnan Qu, Jemaa Essemine, Ming Li, Shuoqi Chang, Tiangen Chang, Gen-Yun Chen, Xin-Guang Zhu

**Affiliations:** 1 State Key Laboratory of Plant Molecular Genetics, CAS Center for Excellence in Molecular Plant Sciences, Shanghai 200032, China; qumingnan@picb.ac.cn (M.Q.); jemaa@picb.ac.cn (J.E.); changtiangen@picb.ac.cn (T.C.); 2 Shanghai Center for Plant Stress Biology, Center for Excellence in Molecular Plant Sciences, Chinese Academy of Sciences, Shanghai 200032, China; mingli@psc.ac.cn; 3 State Key Laboratory of Hybrid Rice, Hunan Hybrid Rice Research Center, Changsha 410125, China; changshuoqi@126.com; 4 Laboratory of Photosynthesis and Environmental Biology, Shanghai Institute of Plant Physiology and Ecology, Chinese Academy of Sciences, Shanghai 200032, China; chenggy@sibs.ac.cn

**Keywords:** dark respiration, CRISPR/CAS9, GWAS, *LRK1*, rice population, molecular genetics

## Abstract

Respiration is a major plant physiological process that generates adenosine triphosphate (ATP) to support the various pathways involved in the plant growth and development. After decades of focused research on basic mechanisms of respiration, the processes and major proteins involved in respiration are well elucidated. However, much less is known about the natural variation of respiration. Here we conducted a survey on the natural variation of leaf dark respiration (*R*_d_) in a global rice minicore diversity panel and applied a genome-wide association study (GWAS) in rice (*Oryza sativa* L.) to determine candidate loci associated with *R*_d_. This rice minicore diversity panel consists of 206 accessions, which were grown under both growth room (GR) and field conditions. We found that *R*_d_ shows high single-nucleotide polymorphism (SNP) heritability under GR and it is significantly affected by genotype-environment interactions. *R*_d_ also exhibits strong positive correlation to the leaf thickness and chlorophyll content. GWAS results of *R*_d_ collected under GR and field show an overlapped genomic region in the chromosome 3 (Chr.3), which contains a lead SNP (3m29440628). There are 12 candidate genes within this region; among them, three genes show significantly higher expression levels in accessions with high *R*_d_. Particularly, we observed that the *LRK1* gene, annotated as leucine rich repeat receptor kinase, was up-regulated four times. We further found that a single significantly associated SNPs at the promoter region of *LRK1*, was strongly correlated with the mean annual temperature of the regions from where minicore accessions were collected. A rice *lrk1* mutant shows only ~37% *R*_d_ of that of WT and retarded growth following exposure to 35 °C for 30 days, but only 24% reduction in growth was recorded under normal temperature (25 °C). This study demonstrates a substantial natural variation of *R*_d_ in rice and that the *LRK1* gene can regulate leaf dark respiratory fluxes, especially under high temperature.

## 1. Introduction

Plant respiration (*R*) is a major element of the global ecosystem, which couples the production of energy to the release of CO_2_. At the ecosystem level, plant *R* contributes up to 65% in the total CO_2_ released into the atmosphere [1]. Globally, terrestrial plants *R* releases about 64 Gigatons of carbon per year (Gt C year^−1^) into the atmosphere [2,3,4]. This is a large flux, compared to the relatively small release of CO_2_ from the use of fossil fuels and cement production (~5.5 Gt year^−1^) and changing land use (about 1.6 Gt C year^−1^) [5]. Therefore, an increase in *R* in response to the climate warming may have a substantial impact on atmospheric CO_2_ concentration, and hence, the greenhouse effect.

Up to two thirds of daily photosynthetic carbon gain is released back into atmosphere by plant dark respiration (*R*_d_) [6,7,8]. Light and temperature are two principal environmental factors that influence *R*_d_. Under light, adenosine triphosphate (ATP) and reducing equivalents (NADPH) are generated in the chloroplast through photosynthesis, which are then used for carbon and nitrogen assimilation. The resulting products of these assimilatory reactions are transported to the cytosol where they are either exported to distant sinks or used to produce ATP and carbon skeletons through glycolysis, tricarboxylic acid (TCA) cycle and the coupled reactions of the electron transfer chain, oxidative phosphorylation [8]. By contrast, in the dark, respiration provides ATP and the metabolic intermediates necessary for growth, maintenance, transport and nutrient assimilation. The interactions among photosynthesis, respiration and photorespiration largely determine the plant physiological properties [9,10,11].

The changes in *R*_d_ under different light environments are partially attributed to the variation in the accumulated carbohydrate and alternative oxidative (AOX) [12]. For example, during daytime, carbohydrate accumulates in mature leaves, while during the nighttime, starch in chloroplasts and fructan in vacuoles break down. The produced sugars and sugar phosphates enter respiratory pathways in several forms, such as fructose-6-P during gluconeogenesis [13]. This process might lead to an enhanced respiration late during the night or early in the morning, which has been called ‘morning rise’ in mature rice leaf [14]. AOX is an enzyme involved in an alternative oxidative pathway, and represents one quinol-oxidizing enzyme in plant respiratory chain and appears to act as an energy overflow pathway when the carbohydrate levels are elevated at the beginning of a night [15,16].

Respiration is classified into growth or maintenance respiration to help differentiate the usage of respiratory-derived ATP for macromolecule biosynthesis in growing versus maintenance of house-keeping functions in full-grown tissues [17]. Generally, growth respiration is associated with growth processes such as the synthesis of new products during growth, nutrient uptake, nitrogen reduction and phloem loading, whereas maintenance respiration is coupled to protein and membrane turnover and maintaining the ions concentrations and gradients [18]. The fraction of daily fixed carbon that comes from respiration varies from 20 to 80% depends on the species, with around half of whole-plant respiration taking place in leaves using various methods through defining different respiratory components from growth, maintenance and ion uptake [16,19,20,21,22]. Thus far, there have been fewer studies on the variation in *R*_d_ within the same species and less knowledge and data availability on the genetic basis of the natural variation in *R*_d_.

The genome-wide association study (GWAS) has recently emerged as a powerful tool to dissect (or decipher) the genetic basis for many important traits in crops [23,24,25]. This method employs a population of accessions representing a broad genetic variability. Notably, we used a minicore diversity rice panel derived from global 97 countries [11]. This panel has diverse evolutionary recombination events [26] and has a high-resolution single-nucleotide polymorphism (SNP) map [27]. Moreover, we used the gas exchange method to measure *R*_d_ [13]. In the current study, we conducted a systematic survey regarding the *R*_d_ in rice and further used GWAS to identify the potential genetic mechanisms controlling *R*_d_. The aim of our investigation is to evaluate the genetic variability of *R*_d_ in rice, characterize the potential genetic loci controlling this variation in *R*_d_ and identify the possible candidate genes underlying this natural variation in *R*_d_.

## 2. Results

### 2.1. Genotypic Variation in R_d_

**Figure 1:**
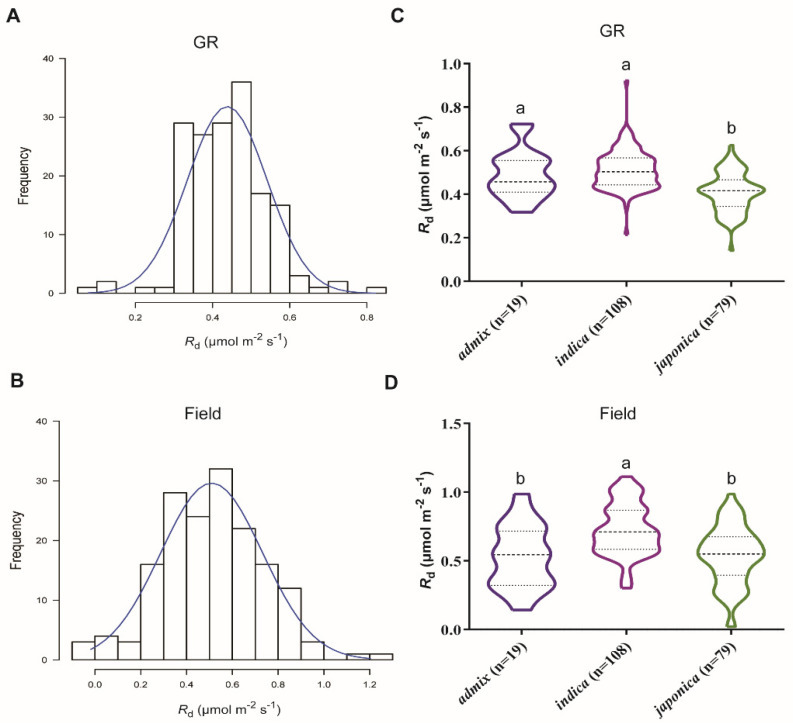
Natural variation of dark respiration (*R*_d_) in 206 rice lines in a global rice minicore diversity panel. (**A**,**B**) Histogram of *R*_d_ under growth room (GR) and field conditions, respectively. (**C**,**D**) Frequency distribution of observed *R*_d_ under GR and field, respectively. *indica* includes *Australica* (AUS) and *indica* (IND); while, *japonica* encompasses *temperate japonica* (TEJ), *tropical japonica* (TRJ) and aromatic (ARO). Admix represents a mixture of *indica* and *japonica*. Different letters are significantly different at 5% level among subpopulations with the Tukey test.

We investigated the nighttime dark respiration (*R*_d_), as well as other leaf physiological traits, including photosynthetic rates under either high (*A_high_*) or low light (*A_low_*), chlorophyll content (SPAD), and leaf thickness (Thickness) in 206 rice accessions of the minicore diversity panel under growth room (GR) and field conditions. Results revealed that a huge natural variation in *R*_d_ under both growth conditions and the variation in *R*_d_ follows a normal distribution (Shapiro-Wilk; *P* < 0.05). The values of *R*_d_ ranged between 0.1–0.9 and 0.08–1.3 μmol × m^−2^ × s^−1^ in GR and field, respectively (Figure 1A,B). The mean values of *R*_d_ across the rice minicore population were 0.46 and 0.58 μmol × m^−2^ × s^−1^ for GR and field, respectively (Table S2). A significantly high SNP heritability (*h*^2^_SNP_) for *R*_d_ in GR was observed (0.48), but not in the field, which was less than 0.001 (Table S2), implying that the presence of a strong genotype-environment interaction since the environment in the field is heterogeneous. The *indica* subpopulation including 108 accessions exhibited relatively higher *R*_d_ than the *japonica* subpopulation constituted with 79 accessions under both conditions, i.e., GR and field (Figure 1C,D).

We then compared the Pearson correlation coefficients (*r*) of *R*_d_ with *A_high_*, *A_low_*, SPAD, and thickness under both growth conditions. Our results show that, in GR, *R*_d_ was positively correlated with *A_high_*, SPAD and leaf thickness (*P* < 0.05), but was negatively correlated with *A_low_*, while in the field condition, *R*_d_ has no correlation with *A_high_*, *A_low_*, and SPAD, except the leaf thickness (Table S3). The *r* values between *R*_d_ with these four traits under GR ranged between 0.15–0.23. A strong positive correlation was observed between *R*_d_ recorded in GR and *R*_d_ in field (Table S3). *R*_d_ variance was significantly affected by either genotype or environmental factor or genotype-environment interactions (*P* < 0.001) (Table S4).

To uncover the effects of natural habitats on *R*_d_, we compared the correlation between *R*_d_ and the geoclimate information of the region from where each accession was collected. Our obtained results show that *R*_d_ for both growth conditions (GR and field) was positively correlated with the annual solar irradiance parameters such as total PAR, diffuse PAR, and direct PAR, annual temperature regimes including minimal and mean temperatures, and annually minimal local dew point (Table S5).

### 2.2. Genome-Wide Association Study on R_d_

**Figure 2:**
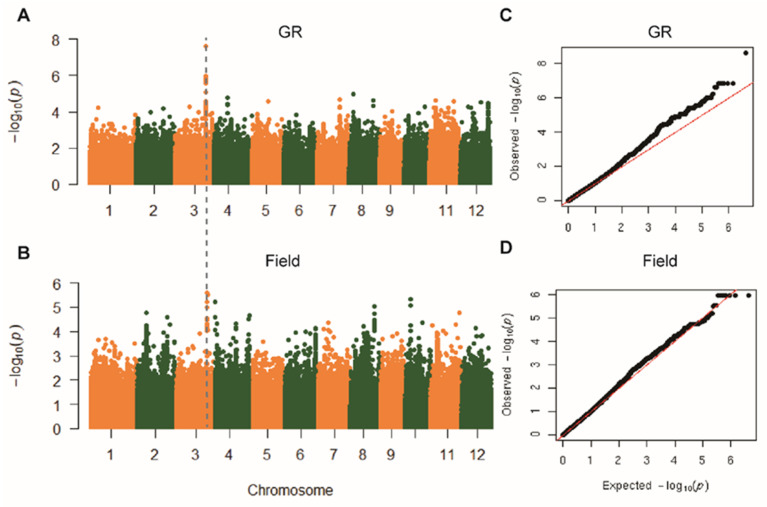
Genome-wide association study (GWAS) performed on the *R*_d_ trait under GR and field conditions. (**A**,**B**) Manhattan plots from the associate mapping of *R*_d_ using a linear mixed model (MLM) in GR and field, respectively. (**C**,**D**) QQ plots of expected versus observed *P-*values of *R*_d_ in GR and field, consecutively. The vertical dashed-line crossing both Manhattan plots at Chr.3 represents the overlapped genomic regions of significantly associated SNPs for both conditions (GR and field).

**Figure 3:**
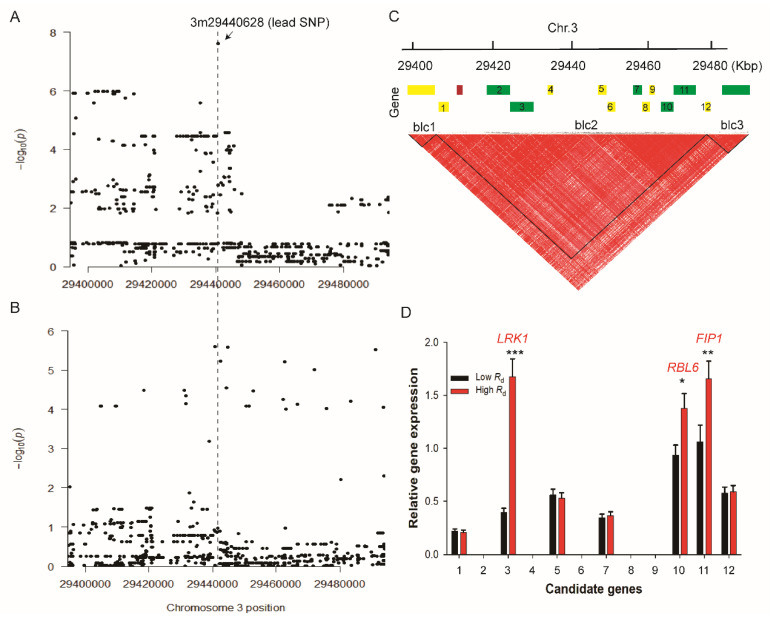
Haplotype analysis of the genomic regions surrounding the lead SNP. (**A**,**B**) Zoom-out of Manhattan plot of the *R*_d_ trait to show the overlapped genomic regions in GR and field conditions, respectively. The vertical dashed-line represents the lead SNP position under both conditions (GR and field). (**C**) LD block covering 100 kb genomic regions centered the lead SNP (3m29440628) in GR. Three blocks (blcs) were obtained within this region. The numbers from 1 to 12 indicate *EP1, SEC14, LRK1, EP2, EP3, EP4, MAF1, EP5, EP6, RBC6, FIP1,* and *EP7*, respectively. Details about genes ID according to Michigan State University (MSU) Rice Genome Annotation Project Database (http://rice.plantbiology.msu.edu) are depicted in Table S1. Expressed, hypothetical and annotated proteins were highlighted in yellow, red, and green, respectively, according to the rice MSU database. (**D**) Relative gene expression levels of the 12 candidate genes against the housekeeping gene, actin1, in two groups of accessions, i.e., the group with high *R*_d_ and the group with low *R*_d_, selected from the rice minicore panel. These 12 accessions with contrasting *R*_d_ values include six accessions having high *R*_d_ and six accessions with low *R*_d_. The detailed genes expressions levels for 12 accessions are displayed in Table S8. Data are means of three biological replicates performed on three different leaves obtained from different plants ±SE.

A genome-wide association study (GWAS) was then performed to identify loci underlying the genetic regulation of *R*_d_. We applied an MLM method, which takes into account the population structure and therefore minimizes false positives rates compared to a general linear model, GLM [28]. A threshold of −log_10_ *P* > 6.0 was determined by 200 times permutations to detect a significant association [27]. There are 24 and 10 significantly associated SNPs for *R*_d_ collected from GR and field, respectively. These SNPs were termed as sigSNP (Table S6). We found that an overlapped genomic region on Chr. 3 for the identified sigSNPs from GWAS based on data from GR and field conditions (Figure 2A,B). The quantile–quantile (QQ) plots shows the upward deviation from the linear line occurred at around −log_10_*P* = 8 and −log_10_*P* = 6 in GR and field, respectively (Figure 2C,D). The lead SNP identified based on data from GR is 3m29440628 with a *P-*value of 2.46 e^−8^ (Table S6). In addition, we characterized some other sigSNPs specific for the field condition, including 4m151738, 10m5015229, 10m5015591, and 8m22334703 (Table S6). The ~100 kb genomic region surrounding the lead SNP was used to screen candidate genes following [23]. The overlapped genomic region identified based on *R*_d_ analysis between GR and field was shown in Figure 3A,B to compare the promising identified genes associated with *R*_d_ measured under both growth conditions. Within this region, there are 16 genes were selected according to the Michigan State University (MSU) Rice Genome Annotation Project Database (http://rice.plantbiology.msu.edu) (Table S7). Based on the haplotype analysis, we found that 12 candidate genes are in the same LD block based on D′ values, with lead SNP (Figure 3C; Table S8).

To further examine the expression patterns of these candidate genes, we compared the differential expression levels at night (11:30 pm) in 12 accessions with contrasting *R*_d_ values (highest and lowest). We used six accessions with high *R*_d_ and six others (accessions) with low *R*_d_ from the minicore population (Table S9). Consistently, we found that the three genes showed significantly higher expressions in the six accessions with high *R*_d_ (Figure 3D, Table S9). Notably, the gene expression level of *LRK1* (Os03g51440) was up-regulated four-fold in these high *R*_d_ accessions (Figure 3D, Table S9). The remaining two genes are *RBL6* (Os03g51510) and *FIP1* (Os03g51520), with a moderate increase in their expression levels by about 0.6-fold (Figure 3D). In addition, we also identified some other genes associated with the specific sigSNPs for the field condition on chromosomes 4, 10, and 8 (Table S10). Some of these genes are annotated to be involved in photophosphorylation process, including *PRC1* (Os08g35420) and *CytoP450* genes (Os08g35510) or in carbon metabolic pathway, such as *STPF1* (Os08g35440), *SDMT* (Os10g09360), *PGM1* (Os04g01230), and *SERP* (Os04g01240), although all of these genes had lower sigSNPs peaks (Table S10).

### 2.3. Haplotype Analysis of Candidate Genes Underlying R_d_

**Figure 4:**
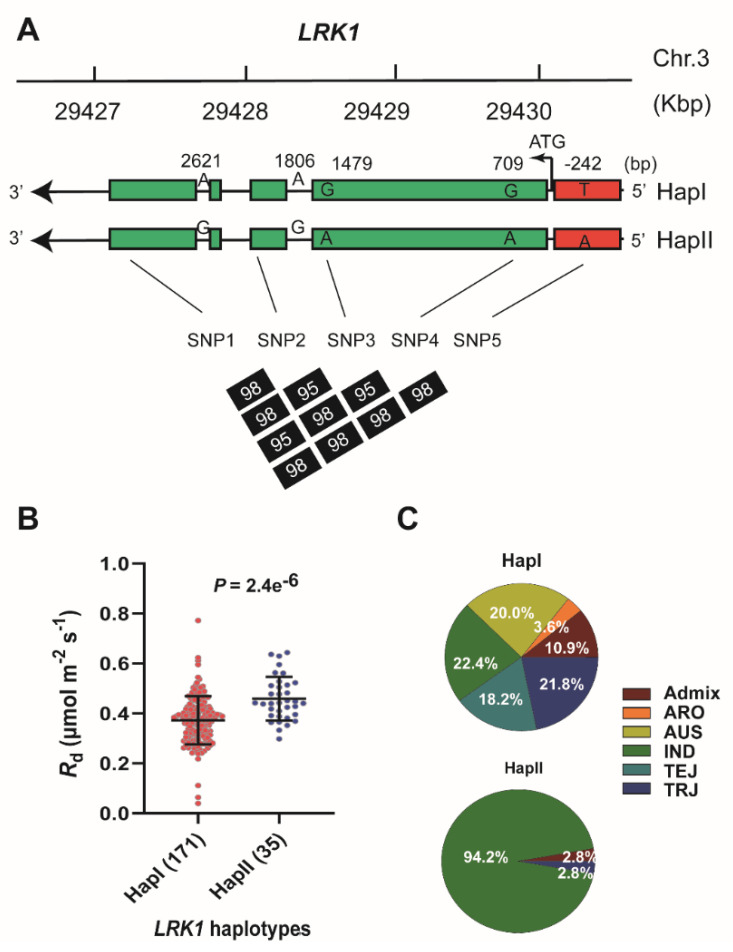
Haplotype analysis of *LRK1*. (**A**) Pair-wise LD between SNP markers on the promoter and coding region of *LRK1* on Chr. 3. The LD was indicated as D′ values via Haploview software. Two haplotypes (HapI and HapII) were shown harboring five alternative SNPs at different position. The five SNPs from SNP1 to SNP5 are 3m29427828, 3m29428645, 3m29428974, 3m29429743, and 3m29430694 at +2621 bp, +1806 bp, +1479 bp, +709 bp, and −242 bp, respectively. SNP1 and SNP2 are located in intron, while SNP 3 and SNP4 are at exon of *LRK1* gene body; however, SNP5 is on promoter region. (**B**) Comparison on *R*_d_ between HapI and HapII with the accessions number for each haplotype are indicated in brackets. A *t*-test was performed to estimate the *P-*value between the two haplotypes. (**C**) Pie-charts of two haplotypes. The percentage of each subpopulation relative to the overall population number was also indicated for each haplotype. Subpopulation includes admixture (admix), aromatic (ARO), aus (AUS), indica (IND), temperate japonica (TEJ), and tropical japonica (TRJ).

We then focused on the lead SNP, because it shows the highest statistical significance level (Table S6). The functional allelic variance within the same LD block surrounding the lead SNP was further analyzed for three candidate genes including *LRK1*, *RBL6*, and *FIP1* through haplotype analysis. Our results show that there are two haplotypes in *LRK1* gene containing five SNPs with a *P* value above the genome-wide suggestive threshold (−log_10_*P* = 5) as previously proposed by [29], including the SNP located at promoter region (−242 bp from the ATP start codon). The suggestive threshold was used to determine the association at lower levels to identify the *LRK1* haplotype variation. Haplotype I (HapI) and haplotype II (HapII) consist of major alleles “TGGAA” and minor alleles “AAAGG,” respectively (Figure 4A). Furthermore, we found that the D′ values between these SNPs across the minicore diversity panels ranged from 95 to 98%, suggesting that these SNPs are likely inherited together (Figure 4A) with a high degree of relatedness. There are 171 accessions in HapI, while HapII contains fewer numbers of accessions (merely 35 accessions). Significantly higher *R*_d_ values were observed for HapII accessions if compared to those of HapI ones (Figure 4B). Notably, most the accessions randomly selected, with extremely high *R*_d_ values and high *LRK1* gene expression levels, are identified to belong to HapII (Table S9). In addition, HapI contains all subpopulations with different proportions (ranged from 3.6% to 22.4%) as depicted in Table S9. Interestingly, most accessions of HapII belong to *indica,* accounting for 94% (35 accessions of HapII).

Since the expression levels of *FIP1* and *RBC6* were also significantly altered between the two groups with contrasting *R*_d_ (as mentioned above), we further analyzed the allelic diversity at the promoter region of the two genes. Two SNPs were identified above the suggestive threshold located at 976 and 719 bp from the ATG start codon of *FIP1*, and one additional SNP above the suggestive threshold located at the third exon (Figure S2A). These SNPs were classified into three haplotypes and exhibited strong correlation with D′ values ranging from 94 to 99%. The HapII contains 150 accessions, which represents three and 13 times more than the number of those of HapI and HapIII, respectively (Figure S2B). Furthermore, the mean *R*_d_ value of HapIII accessions is 10% and 40% higher than that of HapI and HapII accessions, respectively (Figure S2B). Similar to HapII in the *LRK1* gene, we found that most accessions belong to *indica* subpopulation (Figure S2C). For the *RBC6* gene, we found two SNPs located at 608 and 230 bp from the start codon, and one SNP above the suggestive threshold located at the intron (Figure S3A). Relatively higher *R*_d_ values for HapI group containing 95 accessions were observed than that for HapII group with 111 accessions (Figure S3B). These accessions belong to different subpopulations encompassed in the minicore diversity panel with various proportions relative to the total accession numbers for either HapI or HapII (Figure S3C).

### 2.4. Biological Function of LRK1 Gene

In the present report, we mainly focused on understanding the biological function of the *LRK1* gene because it shows the largest differential expression levels between the two contrasting *R*_d_ groups. Notably, there might be multiple genes controlling one complex trait. Therefore, *FIP1* and *RBC6* might also be potential candidate genes influencing *R*_d_, though their effect was smaller than *LRK1*. The two genes can be interesting genes to follow up later. The *LRK1* gene, annotated as a leucine rich repeat receptor kinase, belongs to leucine-rich repeat LRKs (LRR-RLKs) gene family based on UniProt database (www.uniprot.org). We found an orthologue gene in Arabidopsis (*AT2G16250*) in the same branch according to the phylogenetic tree analysis (Figure S4). We also identified some orthologues of *LRK1* for other well-known model plants including *Triticum aestivum, Nicotiana tabacum, Sorghum bicolor,* and *Zea mays*, as well as in some diploid C_4_ grasses such as *Sataria italica* and *Sataria viridis*. This corroborates the importance of *LRK1* biological function in *Viridiplantae* species and its (*LRK1*) evolutionarily conserved aspect. However, the biological function of this gene in different species was not yet well understood.

**Figure 5:**
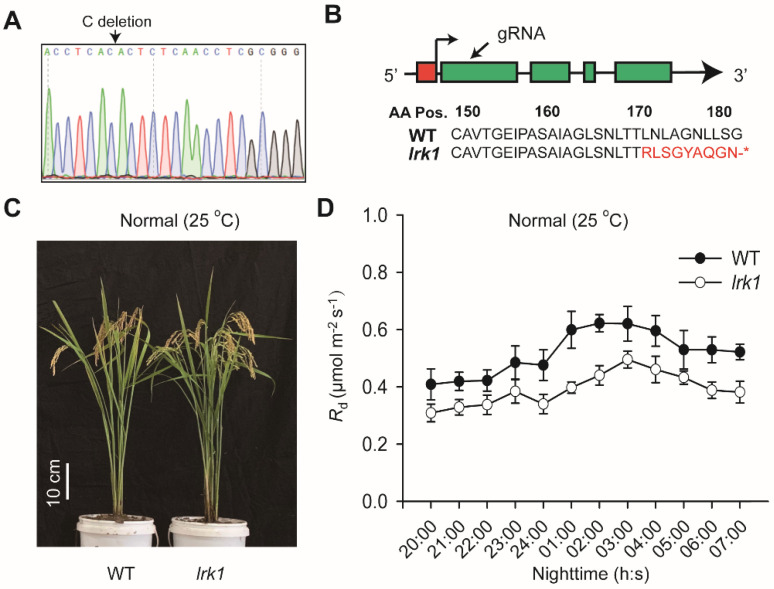
Biological function of knock-out lines of *LRK1*. (**A**) DNA sequencing of CRISPR-CAS9 edited *LRK1*, designated *lrk1*. The arrow indicates the mutation site. (**B**) Target of gRNA in *lrk1*. The arrow indicates the position of guide RNA (gRNA) target. Amino acid (AA) changes due to CRSIPR-CAS9 led to a single “C” allele deletion at 508th nucletide (170th AA), and stop codon at the 180th AA in *lrk1*. (**C**) Performance of WT and *lrk1* mutant grown in GR under ambient temperature (25 °C) and the vertical white bar displays the scaling bar of 10 cm length. (**D**) *R*_d_ dynamics during nighttime in 60-day old plants of WT and *lrk1* mutant grown in GR at 25 °C. Each data point represents the mean of six different replicates performed on different plants ±SE.

Based on GWAS, we demonstrated that the expression of *LRK1* gene is strongly associated with *R*_d_ (Figure 3D). We then analyzed the expression pattern of *LRK1* gene under different light and found that the *LRK1* gene was highly expressed during the nighttime (20:00~07:00), but depressed under daytime especially at noon (12:00) for GR grown WT plants (Figure S5A). Consistently, the expression level of this gene is stimulated gradually after switching to dark from a high light condition (Figure S5B). These findings suggest that *LRK1* gene acts like positive regulator underlying the *R*_d_. Therefore, we used a homozygous rice mutant of this gene edited by CRISPR-CAS9, designated as *lrk1*, against ZH11, a common *japonica* rice cultivar (WT), to analyze the changes in *R*_d_ (Figure 5A). This line has a single “C” allele deletion at 508th nucleotide of *LRK1*, leading to an early stop codon at 180^th^ amino acid (Figure 5B). The mRNA expression between WT and *lrk1* was further confirmed by qPCR, suggesting absence of gene expression in *lrk1* (Figure S6). In the same trend, no phenotypic difference was observed regarding the growth performance of WT and *lrk1* grown in GR under light intensity of ~ 500 μmol × m^−2^ × s^−1^ and 25 °C air temperature (Figure 5C) as well as concerning *A_high_* and *A_low_* (Figure S7A,B), although *R*_d_ was 24% reduced in *lrk1* mutant relative to WT during nighttime (Figure 5D).

**Figure 6:**
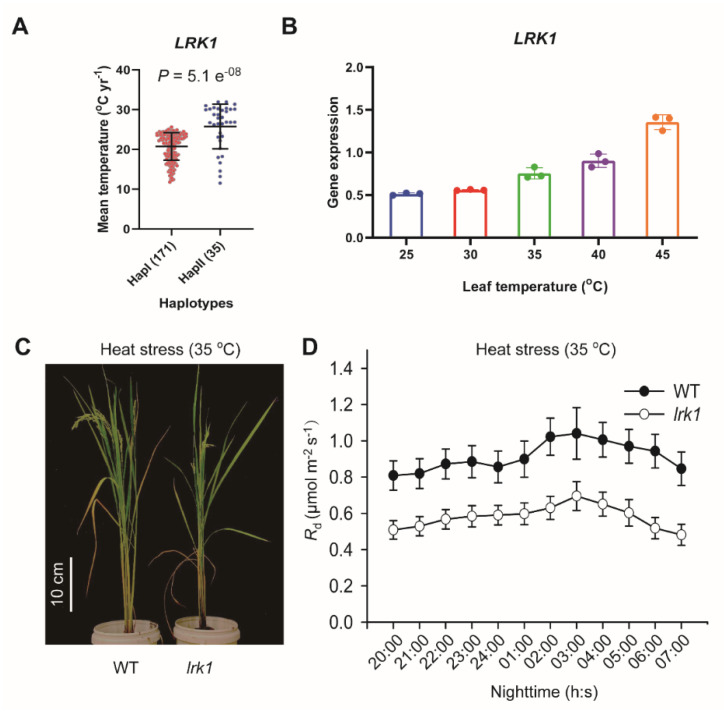
Response of *LRK1* rice mutants to heat stress. (**A**) Comparison of haplotypes of *LRK1* with the mean annual temperature of the regions from where each accession in the minicore panel was collected. (**B**) Temperature-dependent of *LRK1* gene expression levels in 40-day old WT rice plants. Each temperature treatment was applied for 30 min at the leaves level. (**C**) Performance of WT and *lrk1* grown in GR exposed to long-term heat stress (35 °C) for 30 days on 30-day old plants. The white vertical scaling bar represents 10 cm length. (**D**) *R*_d_ dynamics during nighttime for WT and *lrk1* mutant measured at the end of heat treatment (60-day old plants including 30 days at normal temperature and 30 days exposure to 35 °C). For panel (**D**) each data point is the average of six different measurements conducted on six different plants ±SE.

We then compared the geoclimate parameters of the natural habitat with the haplotypes of *LRK1*. In this context, the accessions of HapI show significantly higher values of the mean annual temperature of the regions where accessions were collected than those (mean values) for accessions of HapII (Figure 6A). Our results of the qPCR analysis on 12 rice accessions prove that the accessions with HapII showed high expression levels of *LRK1* (Table S9). We further recorded a gradual increase in the expression level of *LRK1* following an increase in the temperature from 25 °C to 45 °C (Figure 6B), and each temperature treatment was maintained for 30 min.

Ultimately, we applied a long-term (30 days) heat treatment (plus 10 °C relative to normal temperature 25 °C) for both *lrk1* and WT and found that *lrk1* showed retarded growth compared to WT (Figure 6D), which could be potentially related to the dramatic decline in the *R*_d_ in *lrk1* mutant by around 37% if compared to WT (Figure 6D). However, we did not find any substantial change in either *A_high_* or *A_low_* between WT and *lrk1* grown under heat stress condition (Figure S7C,D). The Q_10_, defined as the ratio of *R*_d_ under 35 °C by that under 25 °C [30], of leaf *R*_d_ was dramatically reduced in the *lrk1* mutant compared to WT exposed to heat stress treatment (Figure S8). Our results show also that plant height, tiller number and grain yield per plant were considerably decreased by at least 20% in *lrk1* compared to WT exposed to the same temperature treatment (35 °C), while there was no difference between WT and *lrk1* under normal temperature, 25 °C (Table S11).

## 3. Discussion

The respiratory reactions in metabolic process include glycolysis, the oxidative pentose phosphate pathway, the citric acid cycle, the mitochondrial electron transport chain (mETC), ATP synthase, and several other surrounding reactions [22,31]. The respiration rates can be influenced by genetic, environmental, and/or developmental factors [31,32,33]. However, thus far, the natural variation of respiration and the potential genetic mechanisms underlying and/or controlling this variation is largely unknown. In this report, we studied the leaf respiration rates at night (*R*_d_). Specifically, we used GWAS to identify the functional genes that could be responsible for the variation in *R*_d_, and we found that the variation in the *LRK1* gene, a leucine repeat domain receptor kinase, was strongly associated with *R*_d_ in a rice minicore population.

The fraction of daily fixed carbon especially in *R*_d_ is considerably important (varying from 20 to 80%, depending on the species), given that half of whole-plant respiration takes place in leaves [20]. Thus, the natural variation in *R*_d_ is not only important for plant productivity, but also for the global carbon cycle. We observed an approximate 10-fold change (maximum/minimum) in *R*_d_ between maximum and minimum values in this rice minicore population (Table S2), suggesting a large natural variation of *R*_d_ in the rice population. Considering that the respiration is a major factor controlling plant light use efficiency [34], the identification of genetic factors monitoring the respiration rates might be useful in the future crop breeding and improvements.

To identify the genetic factors underlying the respiratory flux, we proceeded to the IRGA-gas exchange measurements, which has been proved to be the suitable technical tool to perform a rapid screening of *R*_d_ in a large genetic population [35]. Here, we showed that this IRGA-gas exchange measurement in rice minicore diversity panel constitutes a powerful method to identify the genetic basis underlying the natural variation in *R*_d_. In fact, the difference in *R*_d_ between species has been shown to maintain under the same environment [31], suggesting a strong genetic control over *R*_d_. On the one hand, we showed that the SNP heritability (*h*_SNP_^2^) of *R*_d_ determined herein under GR conditions was 0.48, which was significantly higher than that recorded under field conditions (Table S2), consistent with a strong genetic-environment interaction controlling *R*_d_ (Table S4), as also shown earlier in *Hordeum spontaneum* [36] and in Arabidopsis [22]; on the other hand, we observed a strongly positive correlation between *R*_d_ values recorded under GR and field conditions (Pearson correlation coefficient, *r* = 0.4), which implies is the presence of a strong genetic control over natural variation of *R*_d_, which sustains under different conditions (Figure 3A,B).

Based on the GWAS analysis, we demonstrated that the *LRK1* variation was tightly associated with *R*_d_ under both GR and field conditions. *LRK1* is annotated as a leucine rich repeat receptor kinase from UniProt database (www.uniprot.org), which functions as transmembrane receptor proteins that ensure communication between cells (cell to cell) and between the cell and its surrounding environments, especially under stressful conditions [37]. LRKs are reported to be involved in the early steps of osmotic-stress signaling [38], plant responses to various environmental and developmental signals [39,40,41]. The molecular mechanism of how LRK influences *R*_d_ is still unknown; however, experimental data shown in this report reveals a new function for *LRK* in the plant primary metabolism, in addition to its earlier reported roles in the signaling events in response to the environmental changes [42].

In fact, we showed that LRK1 influences the respiratory flux under dark (*R*_d_). Earlier, it was also demonstrated that LRK plays a prominent role in processes of some metabolic pathways induced by light such as sucrose and 3-phosphoglyceric acid (3-PGA) [37]. Indeed, we found that *LRK1* gene was highly expressed during darkness while during the daytime its expression levels dropped to about 10% of that under dark (Figure S5A). This robustly confirms the role of *LRK1* in the modulation of nighttime *R*_d_ (Figure 3D). In a canopy, shaded leaves commonly show low *R*_d_ [13,43,44]. Currently, we also showed that there are substantial diurnal variations in *R*_d_ (Figure 6D), and similar results have been reported earlier in this regard [13]. The light regulation of *LRK1* expression might have contributed to these diurnal changes of *R*_d_ as observed in the present report (Figure S5).

In addition to light, we also corroborated the temperature dependency of the *LRK1* function in rice plants. It is well-known that plant respiration is mostly influenced by temperature [45]. Hence, increasing the air temperature causes a substantial increase in the respiratory fluxes at both the leaf and canopy scales [46]. This temperature dependency of respiration might potentially be related to the *LRK1* biological function. Here, we showed that the expression of *LRK1* is increased with increasing the temperature treatment for 30 min each (Figure 6B). Furthermore, the HapII of *LRK1*, which is associated with higher *LRK1* gene expression, mainly exists in the regions characterized by their higher average temperature than lines harboring the HapI (Figure 6A). Altogether, this implies that the temperature can regulate the *LRK1* expression levels, and hence, influence the *R*_d_.

When plants were grown under 35 °C for 30 days, we did not observe any significant change in the photosynthetic CO_2_ uptake rates in the *lrk1* mutant if compared to WT, under either high or low light (Figure S7). Interestingly, the *lrk1* mutant shows a retarded growth relative to WT under such treatment, except normal condition (Figure 5C), concomitantly with a decrease of 63% in the *R*_d_ for the mutant compared to that of WT (Figure 6D). This result raises a possibility that the decrease in the respiratory rate in *lrk1* might have compromised the plant growth in this mutant. Though increased respiration has always been cited as a cost to plant light use efficiency [34], here we showed that maintaining a proper rate of night-time respiration is also required for plants to gain optimal growth. A delicate balance is needed between minimizing respiratory cost to gain higher light conversion efficiency and maintaining sufficient flux through respiration to maintain the normal plant growth.

In summary, we presented a survey of the natural variation of dark respiration (*R*_d_) in rice diversity panel and we found a large natural variation of *R*_d_. Results from GWAS and gene editing experiments demonstrate that the natural variation of *LRK1* gene is a major factor contributing to the natural variation of *R_d_*. We further showed that the expression of *LRK1* is both a light and temperature regulated process, which might have contributed to the observed diurnal changes in *R*_d_ and temperature responses of *R*_d_. Gene editing experiments using the CRISPR-CAS9 technique further demonstrated a damping in *R*_d_, thereby leading to a decrease in the plant biomass production under elevated temperature. *LRK1* represents a first identified regulator of leaf-based dark respiration under darkness, which might be used to identify a potential target to gain the altered respiration under either different light or temperature conditions.

## 4. Materials and Methods

### 4.1. Rice Minicore Population

In total, 206 rice accessions from a global rice minicore diversity panel were used for dark respiration (*R*_d_) measurements. This minicore panel was developed by USDA-ARS from 97 countries [47]. To study the plasticity of *R*_d_ to environments, we performed experiments under both growth room (GR) and field conditions. For the GR condition, three plants were grown in one pot with two pots for each accession. Potted plants were daily watered and standard nutrition was also supplied to avoid any nutrient limitation. Air temperature, photosynthetic photon flux density (PPFD) and humidity were maintained at around 27 °C, 500 μmol × m^−2^ × s^−1^ and 60–70%, respectively. For field experiments, rice seeds were sown on seedbeds in the field after germination in Hainan (E110°02′, N18°48′), China on 30th May 2018. Seedlings were then transplanted into the field at the fourth leaf stage, which occurred on June 25th 2018. Plants were grown in rows with a row distance of 20 cm and plants in the same row were spaced by 20 cm. A local field management practice was settled down in the field for nutrition supply and pesticide application. For measurements of *R*_d_ and the other physiological traits described below, 60-day old plants were used. Prior to *R*_d_ measurements in the field, six plants for each accession were first dug out and transferred to GR for at least 24 h to ensure full acclimation to the same controlled stable environments.

The geoclimatic data of the minicore population, including total photosynthetic active radiation (PAR), diffuse light, direct light and mean air temperature for the original region from where each rice accession was collected, were downloaded from the National Oceanic and Atmospheric Administration database, NOAA (https://gis.ncdc.noaa.gov/) as previously reported in detail [48]. We collected the geoclimatic information for the years during which each accession was released (1914~1997).

### 4.2. Measurements of R_d_ and Leaf Physiological Traits

Nighttime *R*_d_ is not constant and it is reported to enhance late at night or early morning ‘morning rise’ in mature rice leaves [14]. In this study, we measured the *R*_d_ during the time interval, 02:00 pm~05:00 am, during which the respiratory rate reaches its maximum. We used four portable infrared gas-exchange systems (Li-6400XT; LI-COR, Lincoln, NE, USA) to measure *R*_d_ for 206 rice minicore accessions. During the measurements, flow rates in the leaf cuvette were adjusted to around 300 μmol × min^−1^, leaf temperature was maintained at around 25 °C. Field grown plants were adapted to GR for 24 h prior to measurements as mentioned above. Each measurement took about 2 min. All measurements of *R*_d_ for 206 rice accessions were finished within 3 days. Photosynthetic rates under high light (1500 μmol × m^−2^ × s^−1^) and low light (100 μmol × m^−2^ × s^−1^), termed *A_high_* and *A_low_*, respectively, were measured during the time period 9:30 am ~ 12:30 pm on the same dates during which *R*_d_ was measured. To minimize the effects of growth stage differences, we measured photosynthetic parameters from accession 1 towards 206 sequentially for the first and third replicates and then, conversely, from accession 206 towards 1 sequentially for the second and fourth replicates. In total, we conducted four replicates for each rice accession.

Leaf total chlorophyll content and leaf thickness were determined using a SPAD 502 Plus Chlorophyll Meter (Spectrum Technologies, Aurora, IL, USA), respectively. For each leaf, the SPAD values were recorded as the mean of five measurements at different positions of the leaf middle section. In this context, four replicates from four different plants were used to determine both leaf chlorophyll content and leaf thickness.

### 4.3. Heat Stress Treatments

Plants were grown in pots in GR with the PPFD set to be around 500 μmol × m^−2^ × s^−1^, air temperature set to be around 27/25 °C (day/night), terms as 25 °C, and air humidity set to be 60–80%. During the heat stress treatment, an electronic heater (Meiling Ltd., MDN-RD702, Shanghai, China) was used to elevate air temperature to be around 37/35 °C (day/night), terms as 35 °C, for 30 days. Water and nutrients were routinely added to avoid growth limitations.

### 4.4. Genome-Wide Association Analysis

Details of the DNA sequencing dataset for the minicore panel used for genome wide association study (GWAS) were as mentioned in [27]. Briefly, we used ANGSD (version 0.542, UC-Berkeley, CA, USA) to detect SNP [49]. We further filtered out those SNPs whose minor allele frequency (MAF) was less than 5% in the population. After SNP calling, we found that there was about 30% missing SNPs on average for each chromosome. To obtain the complete SNP matrix, these missing SNPs were imputed using Beagle (version 3.3.2, Beagle Team, Seattle, WA, USA) [50]. The imputed genotype dataset was then converted to a binary genotype file (BED) format for association mapping using PLINK (version 1.07, Center for Human Genetic Research, Boston, MA, USA) [51]. In total, 2.3 million filtered SNPs were obtained. We used the GEMMA software, which takes into account both population structure (Q) and genetic kinship (K) [52] with a multivariate linear mixed model (MLM) to perform GWAS. To reduce the effects of population stratification on SNP signal detection, we applied the first four principal components from principal component analysis (PCA) as covariates. The PC1 and PC2 explain 58.4% and 33.1% phenotypic variance of minicore population structure, respectively (Figure S1). The critical threshold of significance for SNP-trait association was −log_10_*P* > 6.0, which was determined by 200 permutations as proposed by [27]. The Manhattan plot and QQ plot for the GWAS results were generated using the R package qqman in R software (v3.5.4, R Foundation, Auckland, New Zealand) [53]. We used linkage disequilibrium (LD) analysis to investigate the degree of association of the candidate genes to the lead SNP via Haploview software (version 4.2, Broad Institute, Cambridge, MA, USA) as demonstrated by [54]. The LD blocks were defined using D′ value as reported previously [55]:

(1) $$D^{\prime }=D/\mathrm{Dmax}$$

When D is positive, Dmax = min[(p1q2) or (p2q1)]; when D is negative, Dmax = min[(p1q1) or (p2q2)]. The p and q stand for the allelic frequencies of two SNPs. D′ represents a relative measure of disequilibrium (D) compared to its maximum [55]. The values of D′ in a block were calculated following “Gabriel et al.” model [54]. In this study, a 95% confidence bound on D′ was generated. The genes identified in the LD block with lead SNP were considered as potential candidate genes associated with *R*_d_.

### 4.5. Transcript Abundance Analysis

To further screen the candidate genes within the LD block, we quantified the gene expression levels using quantitative real-time PCR (qRT-PCR) analysis among the 12 selected accessions with contrasting *R*_d_ values from the whole minicore panel (206 accessions). The total RNA was extracted with ambion PureLink^TM^ RNA mini kit (Thermo Fisher Scientific, Waltham, MA, USA) according to manufacturer’s instruction and was then reverse transcribed to cDNA with SuperScript VILO cDNA Synthesis Kit (Invitrogen Life Technologies, Thermo Fisher). Reaction was performed using SYBR Green PCR Master Mix (Applied Biosystems, Waltham, MA, USA) and samples were analyzed using a Real-Time PCR System (ABI StepOnePlus, Applied Biosystems) with the following cycling parameters: 95 °C for 10 s, 55 °C for 20 s, and 72 °C for 20 s. Rice *Actin1* gene (*Os03g50885*) was used as control. Relative expression of the target gene against *Actin1* was calculated as: 2^−ΔΔCT^ × (ΔCT = CT, gene of interest^−CT^, *Actin1*), as described by [56]. Three biological replicates were used. The primers used for screening the candidate genes expressions are listed in Table S1.

### 4.6. Development of CRISPR-edited LRK1 Lines

To elucidate the biological function of *LRK1* (Os03g51440) identified based on the GWAS analysis on *R*_d_ measured under both GR and field environments, we obtained CRISPR/CAS9-edited *ChSDG* rice lines (*lrk1*) following the method described previously [25] using Zhonghua 11 (ZH11), a commonly used rice cultivar in China, as the wild type (WT). The sequence of guide RNA (sgRNA) for *ChSDG* is as follows: 5′- TCACCACTCTCAACCTCGCGGGG-3′, which was designed by *CRISPR-P* database with high score (0.863, http://crispr.hzau.edu.cn). The resulted mutation causes a single ‘C’ allele deletion at the 508th nucleotide position in the coding sequence of *LRK1*. Homozygous lines were sequenced using specific primers listed in Table S1.

### 4.7. Statistical Analysis

To evaluate the effects of genotype-environment interaction on the variance of *R*_d_ and other physiological traits in these 206 rice minicore accessions, two-way *ANOVA* analysis based on general linear model was conducted using StatView, ver. 5.0.1 (SAS Institute Inc., Cary, NC, USA). GCTA software (version 1.25.2) was employed, and a restricted maximum likelihood analysis was used to estimate the SNP heritability (*h*^2^_SNP_) of *R*_d_ as described by [57]. The *h*^2^_SNP_ was calculated with 2.3 million filtered SNPs as mentioned above in GWAS imputation section.

## Supplementary Materials

The following are available online at https://www.mdpi.com/1422-0067/21/14/4930/s1. Figure S1. Principal component analysis (PCA) on 2.3 million filtered SNPs in a minicore population encompassing 206 rice accessions. Figure S2. Haplotype analysis of *FIP1* (Fip1 motif family protein). Figure S3. Haplotype analysis of *RBC**6* (ribosomal protein L6). Figure S4. Phylogenetic tree of *LRK1* within other species constructed based on the comparison of protein sequences between spices. Figure S5. Light dependent gene expression of *LRK1*. Figure S6. The expression levels of *LRK1* in WT and CRISPR-CAS9 edited rice lines (*lrk1*). Figure S7. Photosynthetic rates assessed in WT and *lrk1* grown under either normal or heat stress conditions. Figure S8. Q_10_ of the leaf *R*_d_ throughout night-time in WT and *lrk1* mutant. Table S1. List of primers used in this study. Table S2.SNP heritability (*h*^2^_SNP_) of dark respiration (*R*_d_) under both growth room (GR) and field conditions. The SNP heritability is calculated as the ratio of the genotypic variance to phenotypic variance. Table S3. Pearson correlation (*r*) of *R*_d_ to other physiological traits under both GR and field conditions. Table S4. Interaction effects of genotype-environment on *R*_d_ under both GR and field conditions. Table S5. Correlation of *R*_d_ with the geoclimate factors of the regions from where each accession of the minicore panel was collected. Table S6. Significantly associated SNPs list with *R*_d_ under both growth conditions (GR and field). Table S7. Candidate gene list for different significantly associated SNPs with *R*_d_ under GR condition. Table S8. D′ values for linkage block sourounding lead SNP (3m29440628) within a 100 kb genomic region. Table S9. qPCR analysis for the 12 selected candiate genes in 12 rice accessions with contrasting *R*_d_ values (six with low *R*_d_ and six with high *R*_d_). Results are shown as the mean ± SE. Table S10. Candidate gene list for different significantly associated SNPs to *R*_d_ trait in field condition. Table S11. Morphological traits in response to heat stress in WT and *lrk1* mutant evaluated under GR conditions.
